# Targeting Biofilms Therapy: Current Research Strategies and Development Hurdles

**DOI:** 10.3390/microorganisms8081222

**Published:** 2020-08-11

**Authors:** Yu Jiang, Mengxin Geng, Liping Bai

**Affiliations:** NHC Key Laboratory of Biotechnology of Antibiotics, CAMS Key Laboratory of Synthetic Biology for Drug Innovation, Institute of Medicinal Biotechnology, Chinese Academy of Medical Sciences & Peking Union Medical College, Beijing 100050, China; JiangYu_WJ@163.com (Y.J.); gengmengxin@126.com (M.G.)

**Keywords:** microbial resistance, biofilm, EPS, enzyme, antibodies, quorum sensing, metabolic, AMP

## Abstract

Biofilms are aggregate of microorganisms in which cells are frequently embedded within a self-produced matrix of extracellular polymeric substance (EPS) and adhere to each other and/or to a surface. The development of biofilm affords pathogens significantly increased tolerances to antibiotics and antimicrobials. Up to 80% of human bacterial infections are biofilm-associated. Dispersal of biofilms can turn microbial cells into their more vulnerable planktonic phenotype and improve the therapeutic effect of antimicrobials. In this review, we focus on multiple therapeutic strategies that are currently being developed to target important structural and functional characteristics and drug resistance mechanisms of biofilms. We thoroughly discuss the current biofilm targeting strategies from four major aspects—targeting EPS, dispersal molecules, targeting quorum sensing, and targeting dormant cells. We explain each aspect with examples and discuss the main hurdles in the development of biofilm dispersal agents in order to provide a rationale for multi-targeted therapy strategies that target the complicated biofilms. Biofilm dispersal is a promising research direction to treat biofilm-associated infections in the future, and more in vivo experiments should be performed to ensure the efficacy of these therapeutic agents before being used in clinic.

## 1. Introduction

Previous work has revealed that the nature and structure of biofilms is one of the reasons behind drug resistance, which also includes nutrient and oxygen availability to the bacterial cells and intrinsic and acquired bacterial resistance [1]. Approximately 80% of chronic and recurrent microbial infections in humans are caused by bacterial biofilms [2]. Being in a biofilm provides microbes plenty of survival advantages, including, but not limited to, the protection of microbes from the host immune system and antimicrobials/antibiotics, water retention, tolerance to desiccation, sorption and storage nutrient, high extracellular enzymatic activity, adhesion to the infection site, and cell aggregation inducing coordination of virulence factor expression via quorum sensing (QS) [3,4,5]. Traditional treatment of microbial infections is by directly targeting the causative pathogens. However, the presence of biofilms elevated the effective concentrations of antibiotics to a much higher level, and microorganisms in biofilms may develop tolerance to antimicrobial agents through metabolic dormancy or molecular persistence programs that cause the recurrence of biofilm infections after a long period of clinical quiescence.

Biofilm recalcitrance is a result of complex physical and biological characteristics with multiple microbial genetic and molecular factors. It often involves multi-species interactions, so the efficacy of treating biofilm infections with antibiotic alone is poor. Recently, many researchers have switched their focus to anti-biofilm agents, expecting to enhance the efficacy of traditional antibiotic therapies through inhibition of biofilms formation and dispersal of bacteria within mature biofilms that releases the biofilm-associated microbes into their more vulnerable, planktonic state. Dispersion of mature biofilm can be divided into two approaches—active dispersal and passive dispersal—both of which can release planktonic bacteria into the environment. Passive dispersal refers to physical dispersion caused by mechanical intervention or external forces, such as toothbrushing or torn down from the main mass by the flow of interstitial fluid. Active dispersal refers to spontaneous dispersal event of biofilm-associated microbes themselves in response to environmental changes such as nutrient starvation, toxic byproducts, bacteriophages, phagocyte challenge, antimicrobial stress, and unfavorable oxygen levels [6]. Active dispersal is a crucial stage within the lifecycle of a biofilm and is conducive to bacterial survival and disease progression. Therefore, in this review, we focus on four main ways to disperse bacterial biofilm, including targeting the extracellular polymeric substance (EPS) matrix, dispersal molecules, targeting QS, and targeting dormant cells, expecting to induce active dispersal event via external interference. Various mechanical dispersion methods that are being developed, such as improving debridement techniques and surface modification technologies, are only briefly mentioned in conventional treatment methods, without in-depth discussion.

## 2. Conventional Treatment Methods

Other than piled-up assemblages of clonal cells, microbial biofilms represent a dynamic self-constructed ecosystem with a high degree of heterogeneous and compartmentalized milieu [7]. Since the complicated microenvironment inside the biofilm shares similar characteristics with cancer, many biofilm management strategies being devised in clinic are largely based on approach from cancer treatment—early and aggressive irrigation and debridement for physically removal and local delivery of high and sustained antimicrobial chemotherapy [8]. Current biofilm targeting technologies can be divided into the following two groups: (1) physical-mechanical approaches such as high velocity spray and jet irrigators and (2) surface-coating and eluting substrates. Mechanical removal approaches, including debridement of surgical site infections to remove necrotic tissue, exudates, or dental biofilms, has been applied to remove clinical pathogenic biofilms [7]. Surface-coating and eluting substrates can be impregnated with antimicrobials to prevent biofilm formation, and several antimicrobial metal or inorganic coatings have also been applied in clinic to prevent biofilm formation [9]. However, for treating pre-existing biofilms, laboratory studies show that statistically significant reductions of biofilm may require extended incubation periods with high antibiotic concentrations. In situ releasing is an important approach to solve this problem, since higher localized antibiotic concentrations can be sustained for a longer period of time compared with systemic administration [10,11].

Furthermore, high-speed imaging has provided important information on fluid–biofilm–surface interactions, demonstrating that although a statistically significant amount of biofilm is removed from the area, the biofilm gets fluidized and spreads across the surface [12]. The low success rate of irrigation and debridement treatment in periprosthetic infections can be attributed to the ability to the fluidization of biofilms [7]. Nonetheless, antimicrobial agents can be conveniently supplied when using water-based jets—the fluid doubles can create mechanical forces to act on the biofilms while drugs being delivered to act on pathogens at the same time. Despite clinical therapies discussed above having made some progress, mechanical removal methods still have many limitations, and the long-term existence of biofilms may also induce antibiotic resistance. All these problems indicate the urgent need for scientific researchers to seek other strategies for biofilms dispersal.

## 3. EPS-Targeting Strategies

**Figure 1:**
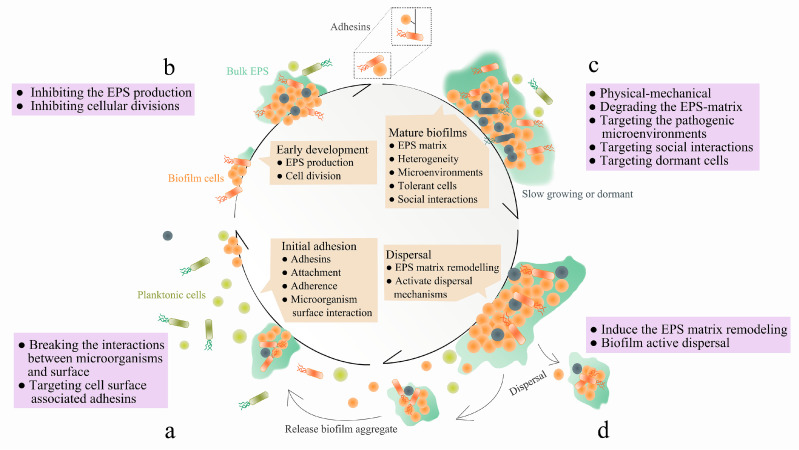
Four stages of biofilm formation: (**a**). “initial adhesion”—microorganisms bind to host or medical device surfaces through cell surface associated adhesins; (**b**). “early biofilm formation”—cells begin to divide and produce extracellular polymeric substance (EPS) to enhance adhesion, while form matrix that embeds the cells; (**c**). “biofilm maturation”—EPS matrix develops 3-D structures which is multi-functional and protective, allowing heterogeneous chemical and physical microenvironments to be formed where microorganisms co-exist within polymicrobial and social interactions; (**d**). “dispersal”—cells leave the biofilm, returning to the planktonic phase. Therapeutic interventions at each stage of the biofilm development. Supplemented and modified based on Figure 1 provided in [7]. Biofilms can be targeted at each stage. (**a**). For example, the initial phase of biofilm formation can be disrupted by breaking the interactions between microorganisms and surface, through targeting cell surface associated adhesins. (**b**). The early stages of biofilm development can be disrupted by inhibiting the EPS production and cellular divisions. (**c**). Mature biofilms can be removed by physical-mechanical approaches, degrading the EPS-matrix, targeting the pathogenic microenvironments and social interactions within polymicrobial biofilms, as well as eliminating dormant cells. (**d**). Induce the EPS matrix remodeling or biofilm active dispersal events.

The development of biofilm includes the following four stages: initial adhesion, early biofilm formation, biofilm maturation, and finally dispersal. Biofilms can be treated at each of the above-mentioned stages (see Figure 1). The EPS matrix is an essential component within these phases, which can promote adhesion to surface, cell-cell adhesion and aggregation [3], and also functions as a 3D scaffold that provides cohesiveness, mechanical stability and protection against host effectors and antimicrobial therapies [7]. In addition, the EPS matrix can dynamically modulate chemical and nutrient gradients, creating pathogenic environments (such as acidic pH and hypoxia) that conduct to the development of key virulence attributes, including recalcitrance [13,14]. Thus, targeting the EPS matrix may be an effective way to remove biofilm, disaggregate bacteria, and disrupt the pathogenic environment [15]. Targeting the EPS matrix can be achieved through the following ways: inhibit the EPS production, block the adhesion through binding to adhesins on the microbial surfaces, and degrade the EPS matrix in mature biofilms.

### 3.1. Targeting EPS Synthesis, Secretion and Adhesins

The synthesis and secretion of EPS is a complicated process with the participation of both signaling networks and non-signaling mechanisms. In general, cyclic-di-GMP and cyclic-di-AMP [16] regulate various EPS-producing exo-enzymes, polysaccharides and adhesins, which are potential candidates to be targeted to inhibit or disrupt EPS [17,18]. For example, glucosyltransferase (GTFs) that controls glucan production in the Gram-positive bacterium *Streptococcus mutans* are regulated by these nucleotide-signaling molecules. Signaling molecule inhibitors which can inhibit the synthesis of Glucan will cause the reduction of pathogenic biofilms accumulation. Using these small molecule inhibitors alone is not superior to the chemicals that are currently in use for biofilms control, like chlorhexidine, but when used in combination, these inhibitors can greatly enhance the therapeutic effects of chemicals [19,20]. Some researchers have also identified several potential small molecule inhibitors of di-guanylate or di-adenylyl cyclase through library screening, or in silico drug discovery combined with bioactivity assessment using in vitro biofilm models [21,22], and has been proved to have potential application value.

Furthermore, inhibitors that target the production of adhesin, and adhesin-binding antibodies or peptides can also break the interactions between microorganisms and host surface. For example, mannosides that target the bacterial type 1 fimbrial adhesin FimH have been demonstrated to prevent catheter-associated urinary tract infections (UTI) in mice by reducing *Escherichia coli* UTI89 colonization and treat chronic cystitis by reducing the *E. coli* EC958 population [23,24,25]. However, the metabolic instability of O-glycoside linkage may result in the low half-life and bioavailability of mannosides, and this can be addressed through replacing O-mannosides with C-mannosides. Experimental results showed that prophylactic treatment with C-mannosides reduced the *E. coli* burden for 2-log’s and treatment of chronic infection resulted in a 4-log reduction in an UTI mouse model [26]. Alkyl-substituted mannose residues can access 100-folds higher affinities to *E. coli* adhesin FimH than mannose [27], and arylmannoside also displays low nanomolar binding affinity to FimH, which is likely due to the hydrophobic interactions with the isoleucine and two tyrosines residues within the binding pocket [28]. These optimizations have improved their affinities significantly, increasing the practicability of potential carbohydrate-based drugs. Similarly, ring-fused 2-pyridones, which can inhibit the biogenesis of curli and type-1 pili, has been shown to reduce the uropathogenic *E. coli* bladder colonization for more than 10-fold in an in vivo mouse UTI model [29]. Other biomolecules that bind to EPS adhesins have been extensively discussed previously [30]. Table 1 summarizes some of the biomolecules as examples.

### 3.2. Targeting EPS Chemical Composition

Biofilms are formed when microbes irreversibly attach to a surface, begin to divide, and provide more diverse adhesion sites to recruit other microorganisms to the substrate [35]. Biofilms are a self-synthesized layer that microorganisms used to protect their microbial communities, consisting of complex polysaccharides, proteins, lipids, and extracellular DNA (eDNA), collectively called extracellular polymeric substance (EPS) [3]. The composition of the EPS matrix is variable both temporally and structurally, depending on the type of microorganisms, local mechanical shear forces, substrate availability, and the host environment [7].

One of the main approaches that bacteria use to achieve active dispersal is to produce extracellular enzymes that act on various structural components of EPS, such as proteins, eDNA, and exopolysaccharides, to promote biofilm dispersion and turn bacteria back to the more vulnerable, planktonic state. Targeting EPS can also destroy the viscoelasticity properties to further weaken the cohesiveness of biofilm and enhance antimicrobial efficacy. Theoretically, clinicians can isolate and purify these enzymes and exogenously apply them to pre-formed biofilms at high concentrations in order to achieve active dispersal. Here, three classes of enzymes that have been studied for biofilms dispersal is reviewed: proteases, deoxyribonucleases, and glycoside hydrolases.

#### 3.2.1. Proteases

Extracellular proteins are major components of EPS that represent a large portion of biofilm’s dry mass [36,37,38,39] and are crucial for microbes to maintain and modify the EPS [40,41]. Certain proteins, such as DNA-binding proteins (DNABPs), functional amyloids/amyloid-like proteins (FA/ALPs), and other biofilm-associated proteins (Baps), are vital contributors to the adhesion property and physical stability of biofilm matrices [6]. Thus, proteases that can degrade EPS extracellular proteins have the potential to disperse a massive biofilm.

A plethora of proteases that are conducive to biofilm dispersal have been identified, such as serine protease Esp secreted by a subset of *Staphylococcus epidermidis*. Purified Esp inhibits biofilm formation and destroys pre-existing *Staphylococcus aureus* biofilms, enhances the susceptibility of *S. aureus* that are embedded in biofilms to an antimicrobial peptide component of the human innate immune system—human beta-defensin 2 (hBD2) [42]. In addition, 2 μg/mL proteinase K can also effectively inhibit biofilm development in bap-positive *S. aureus* V329, as well as other *S. aureus* isolates (SA7, SA10, SA33, SA352), and significantly enhance the efficacy of gentamicin against all *S. aureus* biofilms tested [43]. According to the latest report, cysteine proteases that are secreted by equine mesenchymal stromal cells (MSCs) can destabilize methicillin-resistant Staphylococcus aureus (*MRSA*) biofilms, increasing the efficacy of antibiotics that were previously tolerated by biofilms [44]. Table 2 summarizes some of the proteases that have shown anti-biofilm activities.

#### 3.2.2. Deoxyribonuclease (DNase)

Extracellular DNA (eDNA) functions as a structural scaffold within the EPS in many biofilms, facilitating the bacterial adhesion, aggregation, and horizontal gene transfers [66,67,68,69,70]. Initially, DNA found within biofilms was considered to be leftovers of lysed cells, until eDNA was shown to be a vital contributing component of bacterial biofilms by Whitchurch et al. in 2002 [66], and this finding has also triggered a wave of research trying to destroy biofilms by targeting eDNA with DNases. But in reality, deoxyribonuclease (DNase) has been used to decrease sputum viscosity in cystic fibrosis patients even before the function of eDNA in biofilms was discovered [71]. Current antibiotic treatment of biofilms in the lungs of cystic fibrosis patients is complemented with recombinant human DNase I (rhDNase I) [72]. Mammalian DNase I needs to be glycosylated after translation [73], which precludes the use of cheaper prokaryotic expression systems. rhDNase I used in treatment of cystic fibrosis is produced in mammalian cells, whereas the bovine DNase I commonly used in vitro is purified from bovine pancreas [74]. An active but non-glycosylated version of bovine pancreatic DNase I can be expressed in *E. coli* [75], but glycosylation is required for its full activity and thermal stability [73]. Thus, the scope for using DNase in large-scale biotechnological applications is currently limited by its high cost [76]. DNase I overexpression has been developed in *Pichia pastoris* [77], which may reduce the production cost. It has been shown that bovine DNase I can statistically significantly decrease *Gardnerella vaginalis* colonization on vaginal mucosal epithelial cells in a murine model [78].

Exogenous DNase I is effective against the biofilms of many Gram-negative and Gram-positive bacteria, but its effect is dependent on the age of biofilms [74]. Young biofilms are easily removed, but DNase treatment will no longer be effective once the biofilm ages pass a certain point [74]—for example, 12 h for *S. epidermidis* [79], 80 h for *Pseudomonas aeruginosa* [66], and 72 h for *Vibrio cholerae* [80]. The reason that biofilms get resistance to DNases remains unknown, suggesting that destabilizing biofilms by enzymatic degradation of the extracellular matrix should be combined with enzymes that not only target eDNA, but also polysaccharides and proteins [74]. Table 3 lists some of the DNases that have been demonstrated to have anti-biofilm activity.

#### 3.2.3. Glycoside Hydrolases

Most biofilms are highly dependent on the presence of secreted exopolysaccharides as major EPS constituents [3,88,89]. Exopolysaccharides provide many important functions for the establishment and persistence of biofilms, including, but not limited to, structural stability, physical and chemical defense against antimicrobials and the host immune system, adhesion and aggregation of microbial cells, desiccation tolerance, sorption of organic and inorganic compounds, and providing a carbon source in times of nutrient starvation [3,90,91]. Considering the important role of exopolysaccharides in the establishment and maintenance of biofilm architecture, glycoside hydrolases that targeting exopolysaccharides in biofilms has become a research hotspot.

For instance, the glycoside hydrolases PelA_h_ and PslG_h_ have been demonstrated to be able to disrupt biofilm integrity through degrading the key exopolysaccharides Pel and Psl within biofilm matrix in vitro, respectively. Besides, when treating *P. aeruginosa*-infected wounds, the combination of PslG_h_ with tobramycin will trigger greater bacterial clearance than using tobramycin or PslG_h_ alone. The combination can also improve the innate immune activity, resulting in greater complement deposition, neutrophil phagocytosis, and neutrophil reactive oxygen species production [92]. Although enzyme has the limitations of poor retention and enzymatic stability, which may compromise its efficacy in vivo [93], it has also been reported that the glycoside hydrolase can be used to degrade a mixed-species *S. aureus* and *P. aeruginosa* biofilm grown in a murine model of chronic wounds [93,94,95].

In addition, endolysins, a member of bacteriophage-encoded peptidoglycan hydrolases that can degrade the peptidoglycan layer of bacterial cell wall, has also attracted attention [96]. Engineered peptidoglycan hydrolase constructs with distinct antimicrobial activities have been proved to degrade multiple unique bonds in the *S. aureus*-specific peptidoglycan structure [97] and to increase bactericidal and biofilm removal in animal models. Fusion proteins, which are encoded by multiple bacteriophages and derived from endolysins with unique actions, may reduce the risk of resistance development and show sufficient specificity to avoid targeting commensal strains when supplied simultaneously [7]. Table 4 summarizes some of the glycoside hydrolases with biofilm-disrupting ability.

### 3.3. Targeting Specific Components in EPS and Nucleic-Acid-Binding Proteins

In addition to increasing bacterial resistance to antibiotics, the formation of biofilms also poses challenges to current vaccine treatments. Vaccines are specific to microorganisms; however, clinical isolates from biofilm infections show considerable variabilities in genotype and/or the phenotypic expression of vaccine-targeted epitopes [121]. To address this problem, researchers have developed antibodies that target specific EPS components, such as polysaccharide Psl, which is widely present in *P. aeruginosa* clinical isolates. Currently, monoclonal antibodies against *P. aeruginosa*-derived EPS have been identified with epitopes that bound to the polysaccharide Psl [122]. Anti-Psl antibodies have been shown to have increased opsonophagocytic killing against *P. aeruginosa*, decreased attachment to lung epithelial cells in vitro, and prophylactic protection in multiple animal models of *P. aeruginosa* infection.

In another experimental study, vaccine-elicited antibodies based on *Enterococcus faecalis* pilus tip (EbpA) effectively inhibited biofilm formation in a murine catheter-associated urinary tract infections (CAUTI) model [123]. EbpA, which functions as an adhesin, can effectively block the adhesion of *E. faecalis* to the catheter and inhibit biofilm formation. Notably, it has been found that EbpA did not directly mediate *E. faecalis* adhesion to the catheter material but bind to fibrinogen that deposited on the catheter surface. The results showed that wild-type *E. faecalis* (which expresses EbpA) was not able to adhere to the catheter in vitro and thus was unable to form biofilm, and genes required for biofilm formation in TSGB in vitro were not required in forming biofilms on catheters in vivo [124,125]. This result suggests that biofilm formation model in vitro in TSBG cannot accurately reflect the requirements for CAUTIs in vivo, which highlights the importance to use a more accurate host-microorganism model.

Targeting broadly conserved components in EPS has also been considered as desirable. The DNABII family of DNA-binding proteins, which includes integration host factor (IHF) and histone-like protein (HU), play a key role in providing structural integrity of eDNA [126]. Bacterial biofilms exposed to the antibodies that target DNABII protein will destabilize the eDNA matrix, resulting the collapse of the biofilm structure [126,127]. IHF has specifically been exploited to target nucleoproteins in biofilms due to its high binding affinity and has been widely tested in animal models [7]. Antibodies against *E. coli* IHF are cross-reactive that can bind to DNABII in multiple bacterial species, destabilize biofilms, and release individual bacterium. When combined immunotherapy that targets DNABII with antibiotic therapy, it can be effective against biofilms of numerous types of bacteria in murine lung infection models, including oral bacteria [128], uropathogenic *E. coli* [129], *P. aeruginosa* [127], and *MRSA* [130]. Another approach is to combine DNABII antibodies with vaccines. A study with nontypeable *Haemophilus influenzae* (NTHi) in an animal model of otitis media used IHF and recombinant soluble type IV pili (rsPilA) co-administered with an adjuvant and delivered by transcutaneous immunization to achieve early NTHi eradication and prevention of disease [131]. Recently, a “tip-chimer” immunogen to mimic the DNA-binding regions within the α-subunit and β-subunit of IHF from NTHi (IHF_NTHi_) has been shown to effectively disrupt the biofilm of NTHi in an animal model of otitis media [132]. Besides, antibodies derived against the *Porphyromonas gingivalis* DNABII protein, HUβ, reduce by half the amount of *P. gingivalis* organisms entering into preexisting biofilm formed by four oral streptococcal species, effectively dispersed oral streptococcus biofilm and prevented *P. gingivalis* to enter into oral streptococcus biofilm [133]. Table 5 summarizes some of the molecules that target conserved components in EPS and nucleic acid-binding proteins.

## 4. Dispersal Molecules

Biofilm dispersal is a regulated process involving the degradation of EPS matrix. The triggering of this response provides us a promising research strategy to promote biofilm self-disassembly. Bacteria embedded in biofilms will be more susceptible to conventional antibiotics after their returning to a planktonic state, and the released inactive cells will also lose the protection of biofilms to some degree. Regardless of dispersed state, it remains a vitally important therapy to use dispersive or exogenous EPS-degrading agents alongside systemic antibiotics to avoid recolonization or bacteremia, and potentially septicemia in clinic [7]. Herein, we divide molecules that trigger biofilm degradation into the following four groups: dispersal signals, anti-matrix molecules, sequestration molecules and metabolic interference molecules, collective called dispersal molecules. (See Table 6 for a list of various molecules.)

### 4.1. Dispersal Signals

Many key dispersal signals that can be recognized by microorganisms have been identified, such as the intracellular secondary messenger nucleotide c-di-GMP, which plays a key role in the biofilm lifecycle in both Gram-positive and Gram-negative bacteria, whereby increased levels promote biofilm formation and reduced levels promote biofilm disassembly [138]. Therefore, molecules that can bind free c-di-GMP or regulate enzymes that governing c-di-GMP levels, including diguanyl cyclases (synthesis) and phosphodiesterases (breakdown), are potential dispersal reagents. For example, one study used a *P. aeruginosa* construct containing an exogenous *E. coli* phosphodiesterase to show that reduction in the c-di-GMP level can be achieved via induction of YhjH c-di-GMP phosphodiesterase, which resulted in dispersal of biofilms on silicone implants in a mouse foreign body infection model [139]. Although bacteria accumulated temporarily in the spleen after the induction of biofilm dispersal, the mice were well tolerated to the dispersed bacteria. In another experiment, researchers introduced a functional protein gene *PA2133* containing an EAL domain to degrade c-di-GMP into the modified system, showing that the engineered optogenetic tool inhibited the formation of *P. aeruginosa* biofilms and resulted in much sparser and thinner biofilms, suggesting that the synthetic optogenetic system may be a promising strategy to control and fight against biofilms [140]. Additionally, exogenous nitrate has been shown to be able to reduce the intracellular levels of c-di-GMP and inhibit the biofilm formation of *Burkholderia pseudomallei* 1026b [141].

Endogenously produced nitric oxide (NO) is also a kind of dispersal mediator which can be generated and recognized by both prokaryotes and eukaryotes and are highly conserved [142]. NO was first shown to regulate c-di-GMP levels, mediate biofilm dispersal in *P. aeruginosa* at low concentrations [143], and similar results have been reproduced in several other bacterial species [142]. Glutamate is the second molecule known to induce cells to be released from biofilms and does so in nutrient-induced dispersion process [144]. Some results suggest that biofilm dispersion under both glutamate- and NO-induced conditions may share the same mechanism [145,146]. However, gaseous NO is unstable and has potential cytotoxicity to the exposed host tissues. To address this problem, a cephalosporin-3′-diazeniumdiolates (C3Ds) NO-donor prodrug has recently been developed, which can selectively release NO from the prodrug through contacting with biofilm β-lactamases, allowing improved bacterial killing by conventional antimicrobials at sites of biofilm infections, while also minimizing NO- mediated toxicity [147]. NO-donor instability is also an issue, which is being addressed by developing sterically hindered NO analogues that exert biological responses via NO-mimetic properties [148]. These molecules (carboxy-TEMPO, CTMIO, DCTEIO) trigger biofilm dispersal similarly as NO in *P. aeruginosa* and *E. coli*. The treatment with carboxy-TEMPO also reduced bacteria tolerance to ciprofloxacin [148,149], however, it failed to disperse biofilms formed by *MRSA*, which indicates that this approach may be limited to biofilms formed by certain species [7].

### 4.2. Anti-Matrix Molecules

Another class of biofilm dispersal molecules is those that actively target the EPS matrix, also called anti-matrix molecules [6]. Prime examples of this type of molecules are rhamnolipids, which are microbial-synthesized biosurfactants that were first found to be associated with *P. aeruginosa* biofilms [150]. Normal concentrations of rhamnolipid are important for the maintenance of mature biofilms, particularly for fluid channel maintenance and cellular migration, but once the concentrations of rhamnoids get higher than normal levels, they will trigger a series of biofilm dispersal reactions [150,151,152,153].

An additional example of duality of surfactant function in bacterial biofilms is phenol-soluble modulins (PSMs) produced by *S. aureus*. PSMs are surfactant-like peptides that promote biofilm disassembly in the monomeric form by reducing the surface tension [154], however, amyloid-like fibers will be formed when they undergo orderly aggregation [155]. Polyamines, such as spermidine and norspermidine, also have a dual role. Compounds that structurally mimic norspermidine has been demonstrated to effectively inhibit the biofilm formation of *Bacillus subtilis* and *S. aureus* [156], while both spermidine and norspermidine will also induce biofilm formation in some cases [157,158,159]. Besides, four D-amino acids produce by bacteria, including D-tyrosine, D-leucine, D-tryptophan, and D-methionine, can destroy existing biofilms and prevent biofilm forming [160,161]. The disassembly of biofilms by D-amino acids is associated with mislocalization of a cell wall protein YqxM/TapA that anchors amyloid-like fibers to the cell wall [161,162]. D-amino acids have been proved to effectively work on biofilms of *S. aureus*, *P.aeruginosa*, and *B.subtilis* [161,163,164,165], and the robust effects of D-amino acids on biofilms were not associated with cytotoxicity [161,166,167].

### 4.3. Sequestration Molecules

Molecules that inhibit biofilm formation by binding or interfering with other molecules involved in the production or persistence of biofilm are called sequestration molecules. These sequestration molecules may not directly act upon biofilm microbes but reduce the levels of important secondary messengers, metabolites, and nutrients, triggering active biofilm dispersal event [6]. For example, BdcA, a protein produced by *E. coli* that can bind free c-di-GMP, indirectly inhibiting biofilm formation by blocking the biofilm-sustaining cellular pathways and the molecule’s biofilm-producing processes [168,169,170].

Furthermore, lactoferrin, an innate immunity protein, was shown to disrupt *P. aeruginosa* biofilm formation by sequestering Fe (III) from siderophores [171]. Iron is an essential component of many metabolically relevant proteins in living cells, and the maintenance of biofilms requires higher concentrations of iron than planktonic growth [172,173,174]. The functional siderophore system pyoverdin is required for biofilm maturation of *P. aeruginosa*, and the absence of this major iron uptake system will promote biofilm disassembly [172]. A combination of tobramycin with FDA-approved iron chelators deferoxamine or deferasirox can prevent the biofilm formation of *P. aeruginosa* on cystic fibrosis (CF) airway cells, reducing established biofilm biomass on polarized CF airway cells by approximately 90% and reducing viable bacteria in these biofilms by 7-log units [175].

### 4.4. Metabolic Interference Molecules

The potential of exogenous amino acids in the treatment of biofilms has gained great attentions. It has been proved that specific amino acids can affect both biofilm metabolism and development. For example, L-arginine (L-Arg) can modulate pH homeostasis within oral biofilms via neutralizing acids [176], and high concentrations of L-Arg (>5 mM) results in dramatic reductions of *Streptococcus gordonii* biofilm biomass and changes biofilm architecture [177]. Treatment of polymicrobial biofilms comprised of *S. mutans*, *S. gordonii* and *Actinomyces naeslundii* by L-Arg can suppress *S. mutans* growth and results in substantial reduction in insoluble EPS and alters biofilm architecture [178]. L-Arg can destroy biofilms formed by a variety of oral microorganisms [179] and yeast cells [180]. D-arginine (D-Arg) also can work well on bacterial biofilms. It shows that D-Arg can inhibit and dissociate EPS from biofilms when its concentration exceeds 50 mM and 100 mM, respectively, and can change the *P. gingivalis* biofilm structure at relatively high concentrations [181]. In addition to Arg, L-methionine (L-Met) has also been identified as a promising adjuvant to treating *P. aeruginosa* biofilms, which can trigger biofilm disassembly, increasing sensitivity towards ciprofloxacin in a mouse model of chronic pneumonia, and enhancing survival of infected mice [182]. Given the diversity in amino acid utilization between bacterial species, it is unlikely that a single amino acid would have a universal function, however, the importance of amino acid or bacterial metabolism in general, should not be underestimated in the development of future treatment strategies [7].

Iron metabolism also plays an important role in the biofilm formation of several pathogens [172,183,184,185]. As an essential nutrient, iron acquisition is crucial for pathogens to infect the host. And it has been shown that the increased of biofilm formation by *P. aeruginosa* was linked to increased availability of iron [186]. Host defences normally actively sequester iron to limit the growth of infecting bacteria, however, *P. aeruginosa* possesses multiple redundant iron receptor and uptake systems, such as production of the siderophore pyoverdin, an iron-chelating molecule [7]. Thus, molecules that are chemically similar to iron, which can be absorbed by bacteria but are not the same in its function, can be used to inhibit the iron-dependent pathways required for cell growth and biofilm formation. Transition metal gallium (Ga) is chemical similar with Fe that can substitute for Fe in many biologic systems and inhibit Fe-dependent processes. It has been proved that Ga can inhibit biofilm formation and *P. aeruginosa* growth, kill planktonic and biofilm bacteria in vitro, and is also effective in two murine lung infection models, i.e., acute mouse pneumonia model and chronic biofilm lung infection model [187].

## 5. Targeting Quorum Sensing

Bacteria control important developmental processes by sensing and responding to environmental cues. Two widely conserved and important strategies that bacteria employ to sense changes in population density and local environmental conditions are QS and cyclic di-GMP (c-di-GMP) signaling, respectively [211]. C-di-GMP mentioned above can regulate various EPS-producing exo-enzymes, polysaccharides and adhesins, and also functions as key dispersal signals. QS enables bacteria to restrict the expression of specific genes when the population has a high cell density, resulting in phenotypes that are more beneficial. The opportunistic pathogen *P. aeruginosa* uses QS to coordinate the formation of biofilm, swarming motility, exopolysaccharide production, virulence, and cell aggregation [212]. These bacteria can grow within a host without detrimental effect until they reach a threshold concentration. Then, they become aggressive, developing to the point at which their numbers are sufficient to overcome the host’s immune system, and form a biofilm, leading to disease within the host. QS requires the binding of a signaling molecule to its corresponding transcriptional regulator, which activates the downstream transcription of selected targets, and then the production of many virulent determinants in pathogenic bacteria which requires cell–cell communication [7]. Hence, quorum quenching, the process that prevents QS by disrupting signaling, can be achieved by (i) inhibiting the synthesis of signaling molecules, (ii) mimicking signaling molecules and binding to their receptors, (iii) degrading signaling molecules, and (iv) modifying signaling molecules. These molecules with abilities to quench the QS system called quorum sensing inhibitors (QSIs) [213].

QSIs that target the QS system in Gram-negative bacteria and Gram-positive bacteria have been extensively evaluated for their efficacy on clinically relevant bacterial biofilms using in vitro and in vivo models. For example, the QS autoinducer, AI-2, functions as a chemorepellent in *Helicobacter pylori* by regulating the proportion and spatial organization of biofilm cells, and treatment of in vitro biofilms with exogenous AI-2 resulted in both reduction in the proportion of adherent cells and induction of biofilm dispersal [214]. Some of the oils and plant extracts extracted from traditional medicinal plants include QS inhibiting compounds that can subsequently inhibit biofilms [215]. There are three types of quorum-quenching enzymes that possess abilities to degrade QS signals—acetyl homoserine lactone (AHL) acylase, AHL lactonases, and oxidoreductases [216]. The results of a published study revealed that AHL-lactonase from endophytic strain of *Bacillus cereus* VT96 effectively interfered with the production of AHL, thus inhibit the formation of biofilms [217].

QSIs can inhibit biofilm formation and virulence factors synthesis, while do not pose any threat to the DNA replication and cell division of the bacteria, thus chances of resistance development to such compounds are presumably rare [218]. Hence, these compounds are ideally qualified as adjunct therapeutics and could be administered along with an antibiotic to reduce chances of resistance development and also to increase the effectiveness of antimicrobial therapy [218]. However, reduced bacterial loads are often dependent on the strain and biofilm model [219]. Extensive research has been done in this area, showing that not all QS systems control biofilm formation positively [6], and the toxicity of QSIs is also an important limiting factor in its development. Thus, only a selected list of QSIs are summarized in Table 7. There are also articles that discuss the relationship between QS and c-di-GMP signaling pathways in detail and consider that QS systems can be seen as a kind of mechanism that c-di-GMP pathway used to sense environmental information—local cell density [211]. The integration of QS with c-di-GMP allows bacteria to assimilate information about the local bacterial population density with other physicochemical environmental signals within the broader c-di-GMP signaling network [211].

## 6. Targeting Dormant Cells in Biofilms

Inducing biofilm active dispersal processes and antibiotic therapy both require the cells to be metabolically active, however, evidences show that dormant cells or persisters residing within biofilms play a key role for drug tolerance [7]. Antimicrobial peptides (AMPs) represent an approach to treat biofilms independent of the presence of microbial activity. AMPs is a major part of innate defense molecules against infections. According to the data from antimicrobial peptide database (APD, http://aps.unmc.edu/AP/), more than 3000 peptides with antimicrobial properties have been discovered, and 2675 of those have antibacterial activity. In recent years, some AMPs have been found to have anti-biofilm activity, which are called “anti-biofilm peptides.” The first known anti-biofilm peptide is human peptide LL-37, which is able to inhibit and diminish *P. aeruginosa* biofilms at concentrations far below antimicrobial levels [231]. One of the most important advantages of AMPs is that they are widely conserved and therefore attractive as broad-acting antimicrobial agents that may be useful against both bacterial and fungal biofilms [232,233]. Another advantage is that AMPs target respiring cells as well as persisters and dormant populations, which may reduce the potential for bacteria to develop AMPs resistance [7]. Therefore, anti-biofilm peptides are potential therapeutic agents.

Synthetic peptides that modify specific AMP sequences have been designed and showed inhibitory activity and can enhance killing of *P. aeruginosa* biofilms in invertebrate infection models when used together with antibiotics [234]. However, microbial proteases and the binding of AMPs to EPS matrix components or other host molecules may further diminish AMPs potency [235]. To address this, combine AMPs with the approach that targets EPS matrix may further increase both the entrance and permeabilization properties of AMPs once in the biofilm [232,236]. Although using AMPs to target tolerant cells is a promising approach, remaining active post across a spatially and chemically heterogeneous microenvironment is an important challenge in vivo [7], and the high cost of AMPs synthesis is a barrier for clinical development and commercialization [236]. Beyond AMPs, antibiotics that used for the treatment of infections caused by slow-growing bacteria, such as rifampin, is an alternative strategy. When used in combination with fosfomycin, rifampin can enhance efficacy in treating foreign body *MRSA* biofilm infections in vivo [237]. Based on the current literature, it can be found that not all of these peptides cause biofilm dispersal by simply penetrating the EPS and killing the microbes [6]. As mentioned above for LL-37, certain peptides cause biofilm destruction at sub-MIC levels, suggesting that they are acting on the EPS, or on the microbe’s ability to form or maintain a biofilm [6]. Anti-biofilm peptides have been extensively studied, so we only summarize some of them in Table 8.

In addition to the above, it is worth to mention DNA cross-linking drugs like FDA-approved anti-cancer drug mitomycin C (MMC) and cisplatin [cis-diamminodichloroplatinum(II)], which has been substantiating clinical applicability against bacterial infections. DNA cross-linking drugs mainly forms intra-strand DNA crosslinks and eradicates persister cells through a growth-independent mechanism [238,239], therefore, it is very meriting investigation as a new approach for the treatment of recalcitrant infections. MMC is the first broad-spectrum compound capable of eliminating persister cells, passively transported and bioreductively activated leading to spontaneous cross-linking of DNA, and also worked as potent bactericide for a broad range of bacterial persisters, including commensal *E. coli* K-12, pathogenic species of *E. coli*, *S. aureus* and *P. aeruginosa* [239]. Cisplatin, which was found a little later than MMC, can eradicate persister cells of *E. coli* K-12, enterohemorrhagic *E. coli*, *S. aureus* and *P. aeruginosa*, and more effective at killing *P. aeruginosa* persister cells than MMC; it is also highly effective against clinical isolates of *S. aureus* and *P. aeruginosa* [238]. It should be noted that some anticancer drugs may have intrinsic toxicity if used as antibacterial agents, hence, further animal tests should be performed in order to find the adequate treatment regimens and doses, and to asses if the combination of such drugs with conventional antibiotics improves bacterial clearance. This is also summarized in Table 8.

## 7. Hurdles to Development

The initiation of biofilms formation involves complex and dynamic interactions among the surface, the microorganism and the EPS. With the formation of biofilms, the adhesive strength and viscoelastic properties make the removal of a biofilm from surfaces difficult, and resident microorganisms become tolerant to antimicrobials [7]. The key point that makes biofilm difficult to remove is that using antimicrobials alone will leave residues of biofilms (including dead cells) for microbial reutilization and promoting the colonization of other microorganisms. For those dispersal agents with no antimicrobial activity, the release of planktonic cells may cause overload burden to the host immune system following a massive dispersal event. Furthermore, it has even been shown that dispersed cells may in fact be more virulent than both biofilm cells and regular planktonic cells [254,255]. All of these highlight the importance of complementary therapeutic strategies that target both EPS matrix and the residing microorganisms.

In addition to the problems discussed above, the ability of a drug to penetrate through existing biofilms should also be considered, as this feature may induce the de novo emergence of antimicrobial resistance owing to bacteria being subjected to sub-lethal antibiotic concentrations and affects potential cytotoxicity. One promising approach may be targeting the pathogenic microenvironment, such as acidic pH, hypoxia, and pathogen-derived metabolites, in order to induce active biofilm dispersal in response to environmental changes. In this way, we can degrade the matrix and kill resident bacteria, eradicating the pathogenic niche with precision and minimal cytotoxicity to surrounding tissues [7].

in vivo studies and clinical trials are extremely limited with the expand of research scope on the use of dispersal agents to eradicate medical biofilms. The majority of studies were conducted in vitro on monospecies biofilms, and it is extremely difficult to extrapolate these results to more complicated, multispecies biofilms in living environments [6]. In addition, inhibitory interactions within the host environment, such as proteolytic degradation or small-molecule inhibition of therapeutic agents, as well as potential host-toxicity when utilizing proteases and other enzymes that may cause collateral damage should also be considered [6]. All of these problems make it particularly difficult to popularize dispersal agents into clinic.

Despite these limitations, research on biofilm dispersal remains a booming and promising field. Dispersal agents can target the EPS on a molecular scale or induce the microbes to actively degrade their own biofilms. Multi-targeted or combinatorial therapies is an important developing field, which may be the core to completely eliminate biofilms in the future. Understanding the potential of biofilm dispersal agents can lead to a better control of biofilm-associate diseases in clinics, as well as addressing issues such as antibiotic resistance, and applicable therapies may come into use in the near future.

## 8. Conclusions

Recurrent infections and high antibiotics/antimicrobials resistance caused by biofilms post great challenges to medical community and health field. Biofilm-associated infections are usually difficult to treat because bacteria within biofilm will be more resistant to antibiotics compared with planktonic cells, and the leftovers following antibiotic treatment will promote the colonization of other microorganisms and lead to repeated infections. Current biofilm removal approaches are purely mechanical, with very few new therapeutic options available in clinics, and it is extremely difficult to eradicate the entire biofilm infections. To address this, clinicians have combined physical-mechanical approaches, such as sharp or hydrosurgical debridement, with antibiotics or antimicrobials, in order to achieve better therapeutic effect. However, even with these interventions, some recalcitrant wounds remain difficult to heal, and antimicrobial resistances are developing. Hence, it is crucial to find novel biofilm dispersal strategies that effectively release microbes from the protection of EPS.

Dispersal agents can disrupt the EPS on a molecular scale or induce the microbes to actively degrade their own biofilms to break the protection of EPS to pathogenic bacteria, and improve the therapeutic effect to biofilm-associate infections. In this review, we discuss the current state of research regarding molecular biofilm dispersal agents from four main avenues, and we are mainly focusing on technologies that have shown efficacy in preclinical trials. In general, the main limitation is the paucity of clinical trials, or even in vivo studies. Thus, even though the dispersal agents are promising to be used in future medical biofilms treatments, progress needs to be made on the translating of in vitro results to the in vivo efficacy.

## Figures and Tables

**Table 1:** Biomolecules that target EPS synthesis, secretion, and adhesins.

Biomolecules That Target EPS Synthesis and Secretion		
Name	Summary	References
2-(4-methoxyphenyl)-N-(3-{[2-(4-methoxyphenyl)ethyl]imino}-1,4-dihydro-2-quinoxalinylidene)ethanamine	A kind of quinoxaline derivative which inhibits extracellular polymeric substance (EPS) synthesis and biofilm formation in *Streptococcus mutans* by selectively antagonizing Gtfs instead of killing the bacteria directly.	[19]
tt-farnesol	Targets the expression of key genes during biofilm formation. Those key genes are associated with exopolysaccharide matrix synthesis (*gtfB*) and exogenous stress modulation (e.g., *sloA*) that are essential for cariogenic biofilm assembly. It has been proved to be effective against *S. mutans in vitro* and in vivo.	[20]
myricetin	Targeting the expression of key genes during biofilm formation in vitro and in vivo. Key genes are associated with exopolysaccharide matrix synthesis (*gtfB*) and exogenous stress modulation (e.g., *sloA*) that are essential for cariogenic biofilm assembly. It has been proved to be effective against *S. mutans* and *Escherichia coli*.	[20,31]
Ring-fused 2-pyridones	A member of curlicides, such as FN075 and BibC6, sharing a common chemical lineage with other ring-fused 2-pyridones termed pilicides. Retain pilicide activities and inhibit both curli-dependent and type 1-dependent biofilms.	[29]
Temporin-GHc, Temporin-GHd	Impede the initial adhesion of biofilm and downregulate the expression of glucosyltransferases biosynthesis genes, having been proved to be effective against *S. mutans*.	[32]
**Biomolecules that Target Adhesins**		
Mannosides	A small molecule inhibitor of type 1 fimbriae adhesin FimH that effectively inhibit the invasion of *E. coli*.	[23,25]
ZFH-04269	4′-[α-d-Mannopyranosyloxy]-N,3′-dimethylbiphenyl-3-carboxamide, a small molecular weight compound which inhibits the type 1 fimbriae adhesin FimH and significantly reduces *E. coli* colonization.	[24]
C-mannosides	Replacing O-mannosides with C-mannosides to improve the half-life and bioavailability of mannosides, which was due to the metabolic instability of O-glycoside linkage. C-mannosides have been proved to effectively reduce the *E. coli* burden. Alkyl-substituted mannose residues also have 100-fold higher affinities to the *E. coli* adhesin FimH than mannose.	[26,27]
Arylmannoside	Arylmannoside displays low nanomolar binding affinity to FimH, which is likely due to its hydrophobic interactions with the isoleucine and two tyrosine resodies within the binding pocket.	[28]
FUD	An inhibitory protein that targets *Candida*-fibronectin interactions by blocking the surface adhesion of *Candida* to halt biofilm formation.	[33]
NDV-3A	A vaccine based on the N-terminus of Als3 protein formulated with alum and has been proved to be effective against biofilm formed by *Candida auris*.	[34]

**Table 2:** Proteases that target extracellular proteins. Supplemented and modified based on information provided in [6].

Name	Summary	References
Esp	A kind of serine protease secreted by a subset of *Staphylococcus epidermidis*. Purified *S. epidermidis* serine protease (Esp) can inhibit biofilm formation and destroy pre-existing *Staphylococcus aureus* biofilms.	[42]
Cysteine Proteases	Cysteine proteases secreted by equine mesenchymal stromal cells (MSCs) has been shown to destabilize methicillin-resistant *Staphylococcus aureus* (*MRSA*) biofilms, thereby increasing the efficacy of antibiotics that were previously tolerated by the biofilms (penicillin/streptomycin), and the equine MSCs secretome can inhibits biofilm formation of various bacteria, such as *Pseudomonas aeruginosa*, *S. aureus*, and *S. epidermidis*.	[44]
Aureolysin (Aur)	A *staphylococcal* metalloprotease that has been shown to disrupt *S. aureus* biofilms by degrading Bap and clumping factor b.	[45,46]
LapG	A protease produced by *Pseudomonas putida* and has been shown to trigger biofilm dispersal event through modification of the outer membrane-associated and exopolysaccharide-binding protein LapA.	[47]
Proteinase K	A highly reactive and stable serine protease with a broad range of cleavage activity that targets peptide bonds which are adjacent to the carboxylic group of aliphatic and aromatic amino acids. It is active against the biofilms produced by a wide range of bacteria strains, including *S. aureus*, *Listeria monocytogenes*, *Staphylococcus lugdunensis*, *Staphylococcus heamolyticus*, *Gardnerella vaginalis*, and *E. coli*, *Heamophilus influenza*, and *Bdellovibrio bacteriovorus*.	[48,49,50,51,52,53,54,55]
Spl	A group of six *Staphylococcal* serine proteases that are involved in *S. aureus* biofilm dispersal, possibly through the cleavage of a cell wall-associated protein EbpS.	[56,57]
ScpA, SspB	Belongs to *Staphylococcal* cysteine proteases and have been shown to disperse *S. aureus* biofilms through degrading unknown target(s).	[45,58]
SpeB	A *Streptococcus pyogenes* cysteine protease which has recently been shown to be involved in in vivo dispersal of *S. pyogenes* biofilms through the hydrolysis of surface proteins M and F1, which are hypothesized to be involved in microcolony formation.	[59,60,61]
SPRE	An endogenous *Streptococcal* protease which results in *S. mutans* monolayer biofilm detachment from colonized surface through releasing the surface protein antigen P1.	[62]
Trypsin	A member of pancreatic serine protease that cleaves peptides at the carboxyl side of lysine or arginine, and actives against biofilms produced by multiple bacterial species, including *P. aeruginosa*, *Streptococcus mitis*, *Actinomyces radicidentis*, *S. epidermidis*, and *G. vaginalis*.	[48,55,63,64]
SspA	A *staphylococcal* serine protease that degrades fibronectin binding proteins and Bap in *S. aureus* biofilms.	[46,65]

**Table 3:** DNases that targets extracellular proteins. Supplemented and modified based on information provided in [6].

Name	Summary	References
DNase I	It has been demonstrated that pancreatic deoxyribonuclease (DNase) can deconstruct the established biofilms of a wide range of microbes, including *P. aeruginosa*, *Vibrio cholerae*, *E. coli*, *S. pyogenes*, *Klebsiella pneumoniae*, *Acinetobacter baumannii*, *Aggregatibacter actinomycetemcomitans*, *Shewanella oneidensis*, *S. heamolyticus*, *Bordetella pertussis*, *Bordetella bronchiseptica*, *Campylobacter jejuni*, *H. influenza*, *B. bacteriovorus*, *S. aureus*, *Enterococcus faecalis*, *L. monocytogenes*, *Candida albicans*, and *Aspergillus*	[74]
λ Exonuclease	A kind of viral DNase that can disrupt established *V. cholerae* biofilms.	[80]
DNase1L2	A human DNase found in keratinocytes that has been demonstrated to degrade the established biofilms of *P. aeruginosa* and *S. aureus*.	[81]
Dornase alpha	A highly purified form of recombinant human DNase I (rhDNase I), which has been demonstrated to be effective against the established biofilms of *S. aureus*, *G. vaginalis* and *Streptococcus pneumoniae*.	[72,78,82,83]
NucB	A bacterial DNase produced by the marine bacterium, *Bacillus licheniformis*, which has been shown to be able to degrade the established biofilms of multiple bacterial species, including *B. licheniformis*, *S. aureus*, *S. epidermidis*, *Staphylococcus salivarius*, *Staphylococcus constellatus*, *S. Staphylococcus lugdunesis*, *Staphylococcus anginosus*, *E. coli*, *Streptococcus intermedius*, *Micrococcus luteus*, and *Bacillus subtilis*.	[84,85,86]
Streptodornase	A *streptococcal* DNase that can disrupt the established biofilms of *P. aeruginosa*.	[87]

**Table 4:** Glycoside hydrolases that target extracellular proteins. Supplemented and modified based on information provided in [6].

Name	Summary	References
Cellulase	A glycoside hydrolase produced by multiple microbes that hydrolyzes the β(1,4) glycosidic linkage, and has been demonstrated to induce the dispersal of biofilms formed by *S. aureus* and *P. aeruginosa*.	[94]
α- mannosidase	An acid hydrolase that is thought to be involved in the turnover of N-linked glycoproteins and has been demonstrated to disrupt *P. aeruginosa* biofilms. However, it has cytotoxic effect on A-431 human epidermoid carcinoma cell lines.	[63,98]
β- mannosidase	Hydrolyzes the terminal mannose residues, which are β(1,4) linked to oligosaccharides or glycopeptides, can disrupt *P. aeruginosa* biofilms. However, it has cytotoxic effect on A-431 human epidermoid carcinoma cell lines.	[63,99]
Alginate lyase	A glycoside hydrolase that degrades the exopolysaccharide alginate, which is common in mucoid *P. aeruginosa* biofilms, causing bacterial cell dispersal and increasing antibiotics’ efficacy and phagocytosis.	[100,101,102,103]
α-amylase	A glycoside hydrolase derived from multiple sources that hydrolyzes α(1,4) glycosidic linkages, mediating the dispersal of mature biofilms of multiple bacterial strains, including *V. cholerae*, *S. aureus* and *P. aeruginosa*.	[94,104,105,106]
Dispersin B	A glycoside hydrolase produced by *A. actinomycetemcomitans*, and has been shown to degrade the polysaccharide poly(1,6)-N-acetyl-d-glucosamine (PNAG) through hydrolyzing β(1,6) glycosidic linkages. This enzyme can effectively act against the biofilms formed by multiple bacteria, including *S. aureus*, *A. actinomycetemcomitans*, *S. epidermidis*, *A. baumannii*, *K. pneumoniae*, *E. coli*, *Burkholderia* spp., *Actinobacillus Pleuropeumoniae*, *Yersinia pestis* and *Pseudomonas fluorescens*.	[107,108,109,110,111,112,113,114]
Hyaluronidase	An enzyme that cleaves hyaluronic acid (HA), a component which has been found to be incorporated into the biofilms formed by multiple pathogens, including *S. aureus*, and *S. intermedius*. When utilized against HA-containing biofilms, biofilms dispersal has been observed.	[115,116]
PelA_h_, PslG_h_	Glycoside hydrolases that can disperse mature biofilms formed by *P. aeruginosa* through hydrolyzing the Pel or Psl polysaccharide, respectively.	[117]
PgaB	Disrupts PNAG-dependent biofilms formed by *B. pertussis*, *Staphylococcus carnosus*, *S. epidermidis*, and *E. coli*, through hydrolyzing PNAG, a major biofilm component of many pathogenic bacteria.	[118]
Ega3	An endo-acting α-1,4-galactosaminidase that has been demonstrated to disrupt biofilms formed by GAG-dependent *Aspergillus fumigatus* and Pel polysaccharide-dependent *P. aeruginosa*.	[119]
Sph3	A retaining endo-α-1,4-N-acetylgalactosaminidase which can hydrolyze galactosaminogalactan (GAG), a cationic polymer of α-1,4-linked galactose and partially deacetylated N-acetylgalactosamine (GalNAc) and has been demonstrated to disrupt biofilms formed by *A. fumigatus*.	[120]

**Table 5:** Targeting specific components in EPS and nucleic acid-binding proteins.

Targeting Specific Components in EPS		
Name	Summary	References
Cam-003	A monoclonal antibody that can bind three distinct epitopes on Psl, and have been demonstrated to block the attachment of *P. aeruginosa* to cultured epithelial cells, to inhibit the adherence or formation of denser biofilms.	[122]
EbpA^Full^, EbpA^NTD^	Vaccine-elicited antibodies based on EbpA which mediates serum antibody response, blocks the interaction between EbpA and host, and inhibits the formation of biofilm.	[123]
Quadrivalent vaccine	It is a vaccine that targets four biofilm upregulated immunogens: SA0037, SA0486, SA0688, and glucosaminidase. The combination of quadrivalent vaccine with vancomycin can significantly reduce *S. aureus* numbers.	[134]
**Targeting Nucleic-Acid-Binding Proteins**		
Antisera	Antisera, which is derived against DNABII proteins, has been demonstrated to be effective against biofilms formed by oral bacteria, *E. coli* and *P. aeruginosa*.	[127,128,129]
TRL1068	A native human monoclonal antibody which has low-picomolar affinity to DNABII homologs from important Gram-positive and Gram-negative bacterial pathogens, and it has been demonstrated to be effective in disrupting biofilms of *MRSA*.	[130]
Anti-IHF_Ec_	Hyperimmune antiserum is derived against purified *E. coli* integration host factor (IHF), and has been demonstrated to be effective on biofilms formed by nontypeable *Haemophilus influenzae* (NTHi) and *Burkholderia cenocepacia*.	[126,135,136,137]

**Table 6:** Biofilm-dispersing molecules. Supplemented and modified based on information provided in [6].

Dispersal Signals		
Name	Summary	References
YhjH	*E. coli* phosphodiesterase that can be induced in vivo, led to the reduction of c-di-GMP and dispersal of biofilms on silicone implants in a mouse foreign body infection model.	[139]
*PA2133*	A functional protein gene containing an EAL domain to degrade c-di-GMP, and can inhibit biofilms formation of *P. aeruginosa*, resulting in much sparser and thinner biofilms.	[140]
Nitrate	Nitrate shows the effect of reducing intracellular levels of the second messenger c-di-GMP and inhibiting biofilm formation of *P. aeruginosa*, *S. aureus* and *Burkholderia pseudomallei*.	[141,188,189]
NO	An endogenously produced dispersal signal which can be generated and recognized by both prokaryotes and eukaryotes and are highly conserved. It has been shown to be involved in the dispersal of biofilms formed by *P. aeruginosa, E. coli*, *Fusobacterium nucleatum*, *Serratia marcescens*, *V. cholerae*, *B. licheniformis*, *Shewanella woodyi*, *Neisseria gonorrhoeae*, *Pseudoalteromonas*, *Vibrio fischeri*, *S. aureus*, *B. subtilis*, *Legionella pneumophila*, *Nitrosomonas europaea*, *P. putida*, *C. albicans*, *Candida tropicalis*, and *Ulva linza*.	[142]
Glutamate	The second molecule known to induce the release of cells from *P. aeruginosa* biofilms, and does so in nutrient-induced dispersion process, and may share the same mechanism with NO-induced biofilm dispersions.	[144,145,146]
C3Ds	A NO-donor prodrug that selectively release NO from the prodrug through contacting with biofilm β-lactamases, and allows targeted enhancement of bacterial killing by conventional antimicrobials at sites of biofilm infections, while also minimizing NO- mediated toxicity. It has been proved to effectively disperse *P. aeruginosa* biofilms in vitro.	[147]
Nitroxides	Sterically hindered NO analogues, which exert biological responses via NO-mimetic properties, and has been proved to induce biofilm dispersal in *P. aeruginosa* and *E. coli*, including carboxy-TEMPO, CTMIO and DCTEIO.	[148,149]
*Cis*-2-decenoic acid (CDA)	A kind of fatty acid cross-kingdom signaling molecule, also known as a diffusible signal factor (DSF), which was originally found to be produced by *P. aeruginosa*. This particular DSF has been shown to trigger the dispersal of biofilms formed by *P. aeruginosa*, *E. coli*, *K. pneumoniae*, *Proteus. mirabilis, S. pyogenes, B. subtilis, S. aureus, C. albicans*, *Salmonella enterica*, and *S. mutans*. It should be noted that other DSFs, such as *Burkholderia* diffusible signal factor (BDSF) [190] and *Xanthomonas* diffusible signal factor (XDSF), have been isolated and exhibited similar inductions of dispersal events [191].	[192,193,194,195]
**Anti-Matrix Molecules**		
Rhamnolipids	A microbial-produced surfactant that, at normal levels, is important for the maintenance of mature biofilms, particularly for fluid channel maintenance and cellular migration. At elevated levels, however, these rhamnolipids have been shown to trigger the dispersal of *P. aeruginosa*, *E. coli*, *S. aureus, B. subtilis*, *M. luteus*, and *Yarrowia lipolytica* biofilms.	[150,151,152,153]
PSM	Surfactant-like peptides that promote biofilm disassembly in their monomeric form, by reducing the surface tension, but form amyloid-like fibers when they undergo orderly aggregations.	[154,155]
Polyamines	Polyamines such as spermidine and norspermidine have been proved to effectively inhibit the biofilm formation of *B. subtilis* and *S. aureus*. However, in some cases both spermidine and norspermidine serve as signaling molecules that induce biofilm formation.	[156,157,158,159]
D-amino acids	D-isoforms of certain amino acids, including D-Leu, D-Met, D-Trp, D-Tyr, and D-Phe, have been shown to cause the disassembly of biofilms through multiple hypothesized mechanisms, including (1) inhibition of genes involved in EPS production; (2) incorporation of D-amino acids into the bacterial cell wall, resulting in the loss of cell-surface fibers which are vital to biofilm formation. D-amino acids have been demonstrated to exhibit efficacy against *S. aureus*, *P. aeruginosa*, and *B. subtilis* biofilms.	[161,163,164,165]
Urea	An amide that is theorized to break down biofilms by disrupting the hydrogen bonds that are vital for EPS mechanical stability, and has exhibited dispersal ability against *S. epidermidis*, *P. aeruginosa* and *K. pneumoniae* biofilms.	[196,197]
Chitosan	A polycationic macromolecule derived from the polysaccharide chitin, and has been shown to penetrate and possibly disrupt biofilms formed by *Cryptococcus neoformans*, *L. monocytogenes*, *P. fluorescens*, *Bacillus cereus*, *S. enterica*, *C. albicans*, and *P. aeruginosa*. It is important to note that it has not been proved that chitosan has any direct effect on the biofilm matrix, and it is possible that the molecule achieves biofilm disruption by penetrating the matrix and acting on the microbes themselves.	[198,199,200,201,202]
**Sequestration Molecules**		
BdcA	A protein that reduces unbound c-di-GMP concentrations by binding to, but not degrading, the molecules, hindering the activation of biofilm-related cellular processes, and has been shown to disperse biofilms formed by *E. coli*, *P. aeruginosa*, *P. fluorescens*, and *Rhizobium meliloti*.	[168,169,170]
EDTA	Ethylenediaminetetraacetic acid (EDTA) is a metal-ion chelator that can sequester EPS-matrix-stabilizing ions, triggering biofilms dispersal of *P. aeruginosa*, *H. influenzae*, *S. epidermidis*, *C. tropicalis*, and *Enterococcus faecalis*.	[203,204,205,206,207,208]
Lactoferrin	An iron-binding protein from the innate immune system which triggers active dispersal through chelating irons, an essential bacterial nutrient and global regulator of a variety of processes, including biofilm development and growth. It has been shown to be effective against *P. aeruginosa*, *E. coli*, *S. aureus*, *E. faecalis* and *S. epidermidis* biofilms.	[209,210]
**Metabolic Interference Molecules**		
Deferoxamine, Deferasirox	FDA-approved iron chelators that have been proved to interfere with bacterial iron metabolism, preventing the formation of *P. aeruginosa* biofilms and reducing established biofilm biomass.	[175]
L-Arg	Exogenous amino acids can affect both biofilm metabolism and development, and it has been proved that L-Arg can effectively disrupt biofilm of *Streptococcus gordonii* and *S. mutans*.	[176,178]
D-Arg	D-Arg can inhibit and dissociate EPS production from biofilm and can alter the *Porphyromonas gingivalis* biofilm structure in relatively high concentrations.	[181]
L-Met	L-Met can up-regulate DNase gene expression and target eDNA components in biofilms. It has been proved to be effective on biofilm formed by *P. aeruginosa*.	[182]
Ga	A transition metal that is chemically similar to Fe, thus it can substitute for Fe in many biologic systems and inhibit Fe-dependent processes. It was shown that Ga can inhibit *P. aeruginosa* growth and biofilm formation and kill planktonic and biofilm bacteria.	[187]

**Table 7:** Molecules that targeting quorum sensing (QS) systems.

Name	Summary	References
AI-2	QS autoinducer, functions as a chemorepellent in *Helicobacter pylori* by regulating the proportion and spatial organization of biofilm cells and has been proved to effectively reduce the proportion of adherent cells and induce biofilm dispersal.	[214]
AIP-I	The autoinducing peptide type I activates a regulatory cascade called *agr* system, resulting in the increased expression of invasive factors, including toxins, hemolysins, proteases, and other tissue-degrading enzymes. It has been proved to effectively disrupt biofilm formed by *MRSA*.	[220]
RIP	RNAIII-inhibiting peptide, a heptapeptide that has been shown to inhibit the biofilms formation of both methicillin-resistant and vancomycin-resistant *S. aureus* and *S. epidermidis*.	[221,222,223,224,225,226]
M64	A small molecule that target the MvfR-regulated QS virulence pathway, which can effectively silence the MvfR communication system, thus blocks *P. aeruginosa* virulence both in vitro and in vivo.	[227]
Cinnamic acid	Acts as a competitive inhibitor for the natural ligands towards the ligand binding domain of the transcriptional activators of the quorum sensing circuit in *P. aeruginosa*, LasR and RhlR. It has been proved to effectively inhibit both the production of the QS-dependent virulence factors and biofilm formation in *P. aeruginosa*.	[228]
Trans 4-(2-carboxy-vinyl) benzoic acid	Cell-free extracts of *Natrinema versiforme* which show QSI properties against *P. aeruginosa* and is efficient for biofilm inhibition.	[229]
SM23	A boronic acid derivative of β-lactamase inhibitor, acting as a powerful inhibitor of *P. aeruginosa* biofilm.	[230]

**Table 8:** Targeting dormant cells in biofilms.

Name	Summary	References
Anti-Biofilm Peptides		
1018	A synthetic, modified form of the cationic antimicrobial peptide bactenecin, which can effectively disrupt the established biofilms of *P. aeruginosa*, *E. coli*, *A. baumannii*, *K. pneumoniae*, *S. aureus*, *Salmonella typhimurium*, and *B. cenocepacia*.	[240,241]
17BIPHE2, GF-17	A 17-amino-acid derivative of human cathelicidin LL-37 which has exhibited efficacy in disrupting *S. aureus* biofilms.	[242]
P60.4Ac	A synthetic peptide derived from human cathelicidin LL-37, which consists of 24 amino acids and has been shown to effectively degrade biofilms formed by *S. aureus*.	[243,244]
BMAP-28	Cathelicidin-derived peptides that can effectively degrade biofilms formed by *S. aureus*, *P. aeruginosa*, and *Stenotrophomonas maltophilia*.	[245,246]
DJK-5, DJK-6	Synthetic protease-resistant peptide, D-enantiomeric, partly works through degrading the (p)ppGpp bacterial stringent response signal. It has been demonstrated to effectively disrupt biofilm formed by *P. aeruginosa*, *A. baumannii*, *S. enterica* and *K. pneumoniae*.	[234,247]
UBBLi30	A kind of bacitracin produced by *Bacillus paralicheniformis* UBBLi30 that can significantly inhibit biofilms formed by *M. luteus* and *MRSA*.	[248]
Pug-1	Pomegranate-derived peptides exhibit both antibacterial activity and anti-adherence activity against *S. mutans* and can inhibit the expression of virulence-associated genes and enzymes.	[249]
CSP	Antibiofilm peptides from *Capsicum baccatum* (red pepper) that can effectively restrict the biofilm formation by *S. epidermidis*, without any antibiotic activity.	[250]
GHaK, GHa4K	Temporin-GHa (GHa) derived peptides that can effectively inhibit the initial adhesion and the formation of *S. aureus* biofilms and eradicate the mature biofilms.	[251]
P5, P6.2	Two synthetic designed AMPs which have the ability to interact with bacterial or eukaryotes membranes. P5 displayed antibiofilm activity on both *P. aeruginosa* and *S. aureus*, while P6.2 only on *S. aureus*.	[252]
B-GR23	A brevinin-2 like antimicrobial peptide with antimicrobial activity against *S. aureus*, can reduce the production of EPS on the planktonic growth of *S. aureus* and inhibit nearly all planktonic bacteria to start the initial attachment of biofilm.	[253]
**DNA Cross-Linking Drugs**		
mitomycin C (MMC)	FDA-approved anti-cancer drug, the first broad-spectrum compound capable of eliminating persister cells, passively transported and bioreductively activated leading to spontaneous cross-linking of DNA, and also worked as potent bactericide for a broad range of bacterial persisters, including commensal *E. coli* K-12, pathogenic species of *E. coli*, *S. aureus* and *P. aeruginosa*.	[239]
cisplatin	FDA-approved anti-cancer drug, cis-diamminodichloroplatinum(II), can eradicates persister cells of *E. coli* K-12, enterohemorrhagic *E. coli*, *S. aureus* and *P. aeruginosa*, more effective at killing *P. aeruginosa* persister cells than MMC, also highly effective against clinical isolates of *S. aureus* and *P. aeruginosa*.	[238]
